# The intergenerational impact of mothers and fathers on children's word reading development

**DOI:** 10.1111/jcpp.14107

**Published:** 2025-01-02

**Authors:** Germán Grande, Tonje Amland, Elsje van Bergen, Monica Melby‐Lervåg, Arne Lervåg

**Affiliations:** ^1^ Department of Education Universityof Oslo Oslo Norway; ^2^ Department of Special Needs Education University of Oslo Oslo Norway; ^3^ CREATE – Centre for Research on Equality in Education University of Oslo Oslo Norway; ^4^ Department of Biological Psychology Vrije Universiteit Amsterdam Amsterdam The Netherlands

**Keywords:** Intergenerational transmission, reading environment, home literacy, reading development, genetic confounding

## Abstract

**Background:**

Numerous studies have investigated the associations between the home literacy environment (HLE) and children's word reading skills. However, these associations may partly reflect shared genetic factors since parents provide both the reading environment and their child's genetic predisposition to reading. Hence, the relationship between the HLE and children's reading is genetically confounded. To address this, parents' reading abilities have been suggested as a covariate, serving as a proxy for genetic transmission. The few studies that have incorporated this covariate control have made no distinction between the HLE reported by each parent or controlled for different skills in parents and children. We predicted children's reading development over time by the reading abilities of both parents as covariates and both parents' self‐reported HLE as predictors.

**Methods:**

We analyzed data from 242 unrelated children, 193 mothers, and 144 fathers. Children's word reading was assessed in Grades 1 and 3, and parents' word reading was assessed on a single occasion. Predictors of children's reading development included literacy resources and shared reading activities.

**Results:**

Children's reading in Grade 3 was predicted by mothers' engagement in reading activities and by literacy resources at home, even after controlling for the genetic proxy of parental reading abilities. The longitudinal rate of change from Grades 1 to 3 was not associated with the HLE or parental reading.

**Conclusions:**

Our finding that parental reading skills predicted children's word reading beyond children's initial word reading underscores the importance of considering genetic confounding in research on the home environment. Beyond parental reading abilities, children's skills were predicted by literacy resources in the home and by how often mothers engage in reading activities with their children. This suggests true environmental effects.

## Introduction

Given the importance of children's cognitive development, it is critical to understand how parents can nurture it. One particularly important aspect is the extent to which parents can nurture children's reading skills, since mastering literacy is important both for academic attainment and societal participation (OECD, 2019). Research on the home literacy environment (HLE) has assumed a causal effect of parental nurturing based on significant associations between the reading environment parents provide and children's reading abilities (e.g., Sénéchal & Lefevre,  2002; Sénéchal, 2006; Sénéchal & Lefevre, 2014). However, parents do not only provide the reading environment but also the genes that influence their child's development (Turkheimer, 2000). Thus, parents can indirectly affect children's reading not only by passing on a genetic predisposition for reading (dis)ability to them but also by promoting literacy activities at home or furnishing their home with books. Consequently, gene–environment correlations can arise and confound the effect of the home environment on children's reading abilities. This is because genetic factors shared by parents and their offspring not only influence children's predisposition to reading development but also parental (reading) behaviors, and hence the literacy environment to which their offspring are exposed (Hart, Little, & van Bergen, 2021; Plomin, DeFries, & Loehlin, 1977).

Twin studies suggest that a large part of the variation in children's reading skills is attributable to the variation in their genes. Depending on age and school grades, genetic factors account for 64%–83% of individual differences in word‐reading skills (Harlaar, Spinath, Dale, & Plomin, 2005; Olson et al., 2011; Samuelsson et al., 2008). Thus, this suggests that parents pass on a disposition for reading development to their children (Swagerman et al., 2017). However, as the home environment siblings (including twins) experience is often highly homogeneous, even twin studies cannot inform us about specific environmental factors that contribute to the individual differences in children's reading development, controlling for genetic transmission (D'Onofrio, Lahey, Turkheimer, & Lichtenstein, 2013).

When it comes to specific environmental effects, several nongenetic studies of the HLE have concentrated on identifying aspects of the reading environment that correlate with children's reading abilities, thereby potentially fostering it. A suggested mechanism for this is shared book reading. Shared reading correlates with early reading skills (Mol & Bus, 2011), and intervention studies show effects from shared book reading on early reading skills (Lonigan, Escamilla, Strickland, & Early Literacy Panel, 2008a, 2008b). Another suggested mechanism is parents directly teaching their children letters and how to read. This indeed correlates with children's early reading skills (e.g., Puglisi, Hulme, Hamilton, & Snowling, 2017; Sénéchal, 2006; Silinskas, Torppa, Lerkkanen, & Nurmi,  2019), and also intervention studies have demonstrated that parental tutoring can have an impact on children's early reading skills (e.g., Lonigan, Escamilla, Strickland, & Early Literacy Panel, 2008a, 2008b). Further, a responsive mechanism is that parents seem to provide more support when children struggle with reading, which suggests that children's (genetically influenced) struggles may evoke reading support (Silinskas et al., 2012; Silinskas, Niemi, Lerkkanen, & Nurmi, 2013). This is an example of evocative gene–environment correlation. A final mechanism is that literacy resources in the home create literacy opportunities for children, thereby enhancing their reading skills (Georgiou, Inoue, & Parrila,  2021; Leseman & De Jong, 1998). Literacy resources are typically simply measured by asking about the number of books in the home. Importantly, in all the nongenetic observational studies, the shared genes between parents and their children are an unmeasured confounder. As a result, it is still unclear whether these consistently observed home environment factors in observational studies genuinely influence children's reading or simply correlate with it due to shared genetics.

One way to reduce genetic confounding when studying the impact of the home environment in observational studies is through the familial control method (Hart et al., 2021; van Bergen, van Zuijen, Bishop, & de Jong, 2017). This method proposes that when direct measurement of genetic transmission is not feasible, one can measure the same heritable trait in the parents as in the child. Thus, using the parental measures as the best available proxy control for the liability each parent passes on. The familial control method is particularly well suited to word reading since word‐reading ability shows reasonable measurement invariance over time and across generations and because the parent–offspring correlation reflects genetic transmission (Swagerman et al., 2017; Wadsworth, Corley, Hewitt, & DeFries, 2001; Wadsworth, Corley, Hewitt, Plomin, & DeFries, 2002). However, if the parent‐offspring correlation were also to reflect environmental transmission—that is, if exposure to a parent with a certain reading ability were to influence children's reading ability—the familial control method would be too conservative as a control and take away some of the environmental variance (Hart et al., 2021; van Bergen et al., 2017).

Studies using familial control methods have demonstrated the genetic confounding effect in the HLE‐child relationship. In the seminal study by van Bergen et al. (2017), parental word‐reading abilities partially explained the concurrent association between literacy resources (e.g., books at home) and child word reading. Controlling for parental reading skills attenuated all the correlations between child reading and the HLE, emphasizing the genetic confounding. Importantly, this study showed evidence of environmental effects since the literacy resources at home and the reading ability of both parents independently predicted children's word reading. A limitation is that this study was concurrent and did not examine parental teaching of literacy activities.

Notably, studies that followed in using the familial control method have found significant associations between the HLE and children's skills. However, while some studies have measured different skills in parents and children (Dulay, Cheung, & McBride, 2019; Puglisi et al., 2017; Zhang, Inoue, Zhang, Jin, & Georgiou, 2024), others have only considered mothers' skills (Puglisi et al., 2017; Zhang, Inoue, & Georgiou, 2023). For instance, Puglisi et al. (2017) used maternal phonological skills (instead of reading skills) as a proxy for the shared genes important for reading skills. This might not offer the same level of control. Even more importantly, using only mothers' phonological skills, and not those of fathers, essentially halves the control for shared genes between children and parents. Controlling for different skills in parents and children may also underestimate the HLE effect if the association between parents' and children's skills also reflects environmental transmission.

Lastly, parental self‐reported reading difficulties have also served as a control for genetic confounding in predicting reading fluency in both middle school (Psyridou et al., 2023) and in adulthood (Khanolainen, Psyridou, Eklund, Aro, & Torppa, 2024). However, it is important to mention that these studies dichotomized parental reading (dis)ability, thereby restricting parental reading variation and, consequently, the confounding control. In addition, parents' reporting of reading difficulties might not be as valid a proxy of genetic confounding as parents’ actual reading skills.

Here, we aim to expand on previous research by including measures of both reading activities and literacy resources. Further, we measure literacy resources more thoroughly by asking both parents to report on the number of books in the home and assessing both parents' knowledge of children's literature (e.g., Hamilton, Hayiou‐Thomas, Hulme, & Snowling, 2016; Puglisi et al., 2017; Sénéchal & Lefevre, 2014), since the number of books alone is susceptible to reliability issues (Eriksson, Lindvall, Helenius, & Ryve,  2022). Taken together, our study explores whether the intergenerational impact on child word reading is purely environmental or whether mothers' and fathers' reading ability confounds this influence, distinguishing the HLE reported by each parent. Our longitudinal design also allows us to examine the intergenerational impact on reading development by controlling for children's earlier word reading.

Thus, we examine the following research questions:

1. Do parents' literacy activities and resources predict Grade 3 reading, over and beyond children's rate of learning between Grades 1 and 3?
2. If predictive, do parents' literacy activities and resources still predict Grade 3 reading, over and beyond the rate of learning and parental reading skills? If so, this suggests a genuine impact of the home literacy environment.

## Method

### Participants

The sample included data from 242 unrelated children and parental information from 193 mothers and 144 fathers. The children's assessments took place in Grade 1 (December 2018–March 2019) and in Grade 3 (November–December 2020). In Norway, formal reading instruction starts at the beginning of Grade 1. The parental information was collected while the children were in Grade 2 (March–June 2020). We initially recruited 259 children (126 girls) in preschool, when they had a mean age of 5.5 years (range 4.9–6.0 years, *SD* = 0.29). Six children with language, behavioral, or cognitive difficulties were excluded from the study. Additionally, five families withdrew from the study, while five moved away from the area. One child was absent at all time points.

Of the 193 participating mothers, 99.5% were biological mothers, and 91.2% had Norwegian as a first language. Of the mothers with a different first language, 2.1% used this as the primary language of interaction with the child. Of the 144 fathers, 98.6% reported being the biological father, and 92.4% had Norwegian as their first language. Of the fathers with a different first language, 4.9% reported using this as the primary language of interaction with the child. We evaluated whether the parents who used Norwegian as the primary language of interaction with their child differed from those who did not in terms of educational level, income, and word reading ability. No significant differences between parents were found, and all the variables were approximately normally distributed (for more details, see the Online Supplement in the study's OSF repository linked below ).

In general, our sample included highly educated parents in comparison to the Norwegian population (Statistics Norway, 2021a), with 87.5% of the mothers and 80.5% of the fathers having completed 1–4 years of university. The annual income level of the mothers and fathers was around that of the female and male populations (Statistics Norway, 2021b).

### Procedure and design

The children attended 61 preschools and 34 schools in municipalities surrounding the eastern parts of the greater Oslo area in Norway, with an average of 4.28 children per preschool and 7.62 per school. Children were assessed in their respective preschools and schools by trained examiners. We collected parental consent for participation in the study and each child's oral assent before their assessment. Families were informed about the possibility of withdrawing from the assessment at any point. The procedures for our study were approved by the Norwegian Agency for Shared Services in Education and Research.

We gathered information about the reading environment directly from the parents on two separate occasions using the Qualtrics© (2020) survey platform. On the first occasion, we sent the fathers and mothers individual text messages (SMSs) linking to a survey optimized for mobile devices. From this survey, we collected background information about the parents (e.g., educational level) and information about the frequency of reading‐related activities at home. On the second occasion, the parents were contacted via phone and provided with links to the digital survey. While on the phone, they were shown titles and authors from children's literature in a recognition task. The nonbiological parents were then guided to the end of the interview, while the biological ones continued with an assessment of word‐reading fluency. Parents were instructed to access the assessments through an online platform and read the word lists aloud. Trained examiners scored the reading fluency over the phone. The completion time of the SMS survey on the first occasion was 2–3 min, while the phone interviews on the second occasion lasted under 15 min. If we only had one parent's contact details, we asked for the other parent's information at the end of the interview.

### Measures

#### Parental literacy activities

##### Parental activities

In Grade 2, each parent was asked how often they performed reading activities other than homework with their child. We asked how often each parent (a) took the initiative to help their child read words (e.g., in daily life, such, as those on cereal boxes or signs in the street), (b) asked their child to read aloud for them, (c) read to their child at times other than bedtime, and (d) read bedtime stories to their child. The parents rated their responses with a 5‐point Likert scale (1: rarely or never, 2: once a month, 3: once a week, 4: several days a week, and 5: most days of the week).

#### Literacy resources at home

##### Books at home

We asked each parent to estimate the number of children's books available at home (0: 0–20, 1: 21–40, 2: 41–60, 3: 61–100, 4: 101–150, 5: 151–200, and 6: more than 200).

##### Title and author checklists

Parents were asked to recognize titles and authors from children's literature. Each list contained real and made‐up items (i.e., foils) to prevent parents from guessing. They were informed about the foils and instructed to select only the items they recognized. They had 3 min, and all completed the task within that time. The title‐recognition checklist consisted of 28 real children's literature titles and 22 foils, while the author‐recognition checklist consisted of 34 real authors and 16 foils. The items were selected from a pool of the most sold and loaned books for children in categories of picture books, child novels, and classics for ages 3–5, 6–8, and 9–13. We corrected the sum scores for guessing by subtracting the number of selected foils from the number of correctly recognized items. The correlation between the title and author recognition checklists indicated good reliability (*r* = .69 and *r* = .64 for mothers and fathers, respectively).

#### Child reading ability

##### Word reading

We measured word reading with a Norwegian translation of the Test of Word Reading Efficiency (TOWRE; Torgesen, Wagner, & Rashotte, 1997) forms A and B with real words. Each child was asked to read words as quickly and correctly as possible within a time limit of 45 s. Each list consisted of words presented in four columns with increasing difficulty, including two columns for monosyllabic words and two for multisyllabic words. We used the number of words read correctly per minute to measure word reading in Grades 1 and 3. The correlations between forms A and B were .94 and .93 for Grades 1 and 3, respectively, reflecting excellent reliability.

#### Parental reading ability

##### Word reading fluency

We expanded the Norwegian translation of the TOWRE (Torgesen et al., 1997) used to assess children in Grades 1–3. We extended forms A and B with pseudowords by adding 17 multisyllabic nonwords with increasing difficulty. In the online survey, each parent was instructed to read the words column‐wise as quickly and correctly as possible within a time limit of 45 s. Then, they were asked to press a start button to activate a full screen with the word list in landscape mode and the exit button to move on to the following reading list. The examiner scored each word simultaneously on a paper version as either correct or incorrect over the phone. The scores were transformed to words read correctly per minute. The high correlation between the two lists indicated high reliability for mothers (*r* = .89) and fathers (*r =* .93).

### Missing data

Missing data analyses using the missing completely at random test (*mcar_test*) of the naniar package (Tierney & Cook,  2023) in R (R Core Team, 2021) revealed 27 missing patterns in the parent–child data (27.8% data missing). The test concluded that the data were missing completely at random (*χ* ^2^ (365) = 328.66, *p* = .914), and therefore, full information maximum likelihood (FIML) was used in all subsequent analyses, giving unbiased results.

### Analysis

To study the intergenerational impact of mothers and fathers on child word reading, we tested each parent's self‐reported literacy activities and literacy resources as predictors of child word reading in Grade 3. Subsequently, we employed mothers' and fathers' word reading abilities as separate predictors of child word reading and correlates of the reading environment. The analyses were estimated using the SEM framework in Mplus Version 8.3 (Muthén & Muthén, 1998‐2017), and the descriptive analyses were performed in R (R Core Team, 2021). The data and output files (Mplus and R Markdown files) are available at https://osf.io/7qcr4/.

First, we estimated the longitudinal change in children's word reading between Grades 1 and 3 with a latent change score model (Geiser, 2020), getting an estimate of the word reading in Grade 3 (intercept) and a change score representing the rate of change between Grades 1 and 3 (slope). Grade 3 reading was regressed on the change score to account for how fast the children learned to read between Grades 1 and 3. Second, we regressed Grade 3 word reading on the measures of the reading environment (activities and resources reported by each parent). The change score was allowed to correlate with the reading environment and parental reading ability. The confirmatory factor analysis with the parental activities and literacy resources is described in Appendix S1. In the final model, we tested if the association between the reading environment and child word reading in Grade 3 changed after controlling for mothers and fathers' word reading.

Last, to account for the possibility that children and parents shared more similarities with other members of the same school cluster, we adjusted the standard errors using the Huber–White estimator (type = complex, cluster = school_ID) in Mplus in our analyses. The adequacy of all the models was evaluated in terms of cut‐off criteria for model fit statistics (Hu & Bentler,  1999).

## Results

The descriptive statistics for all measures are shown in Table 1, and the correlations between the observed indicators in Table 2. All the measures were normally distributed. As the correlations show, child word reading in Grades 1 and 3 was significantly correlated with fathers' title and author recognition checklists (*r* = .22–.31) and with the father‐reported number of books at home. Children's word reading ability was significantly correlated with fathers' (*r* = .22–.29) but not with mothers' word reading ability (*r* = .05–.11). In addition, fathers extra reading was negatively correlated with the children's word reading ability at Grade 3.

**Table 1:** Descriptive statistics of observed scores of parents and child measures

Measure	*n*	*M*	*SD*	Max–Min	Skewness	Kurtosis
Parents						
1. Education_M	193	4.50	1.12	1–6	−0.57	0.10
2. Education_F	144	4.38	1.25	0–6	−0.62	0.05
3. Income_M	193	4.74	1.86	0–9	0.41	0.20
4. Income_F	144	6.45	1.90	2–9	−0.03	−1.14
5. Nr. Books_M	193	3.01	1.51	0–6	0.25	−0.55
6. Nr. Books_F	144	2.62	1.59	0–6	0.27	−0.74
7. Listen the Child Read aloud_M	192	1.76	1.11	0–4	−0.06	−0.79
8. Listen the Child Read aloud_F	144	1.46	1.07	0–4	0.31	−0.46
9. Spontaneous Reading_M	193	2.74	1.13	0–4	−0.69	−0.21
10. Spontaneous Reading_F	144	2.38	1.20	0–4	−0.28	−0.81
11. Extra Reading_M	192	1.23	0.99	0–4	0.41	−0.58
12. Extra Reading_F	144	1.08	1.03	0–4	0.65	−0.45
13. Bedtime reading_M	192	2.57	1.43	0–4	−0.57	−1.06
14. Bedtime reading_F	144	2.22	1.55	0–4	−0.21	−1.51
15. Title Checklist_M	170	8.58	4.07	1–23	0.66	0.32
16. Title Checklist_F	141	5.91	3.59	0–18	0.60	0.46
17. Author Checklist_M	170	10.30	4.60	1–28	0.71	0.59
18. Author Checklist_F	141	7.99	3.92	1–21	0.81	0.39
19. Mother Word Reading A	165	71.85	11.75	36–98	−0.45	0.20
20. Mother Word Reading B	165	72.12	11.30	37–98	−0.37	0.50
21. Father Word Reading A	136	70.12	12.95	36–97	−0.51	0.11
22. Father Word Reading B	136	71.03	13.01	28–96	−0.52	0.42
Children						
23. Child Word Reading_A G1	240	21.81	13.46	0–62	1.00	1.10
24. Child Word Reading_B G1	240	18.92	13.28	0–57	1.21	1.05
25. Child Word Reading_A G3	229	63.53	17.81	22–102	−0.06	−0.78
26. Child Word Reading_B G3	229	64.22	19.16	14–104	−0.28	−0.32

F, fathers; G1, Grade 1; G3, Grade 3; 5–6: number of books at home; 7–8: asking the child to read aloud: 9–10: helping the child read words in daily life (e.g., cereal box); 11–12: reading to the child at different times than bedtime; 13–14: reading at bedtime; 15–18: title and author recognition checklist; 19–22: parents' nonword reading fluency tasks (TOWRE A & B); 23–26: children's word reading fluency tasks (TOWRE A & B); M, mothers.

**Table 2:** Pearson's correlations between observed indicator scores

Measure	1.	2.	3.	4	5.	6.	7.	8.	9.	10.	11.	12.	13.	14.	15.	16.	17.	18.	19.	20.	21.
1. Nr. Books_M	—																				
2. Nr. Books_F	.55***	—																			
3. Listen R_M	.11	.13	—																		
4. Listen R_F	−.07	.15	.27***	—																	
5. Spontaneous R_M	.23***	.17*	.47***	.12	—																
6. Spontaneous R_F	.03	.18	.17*	.54***	.21**	—															
7. ExtraR_M	.04	.17	.40***	.13	.33***	.01	—														
8. ExtraR_F	.10	.21	.16	.51***	.04	.36***	.05	—													
9. Bedtime_M	.21**	.19**	.28***	.01	.37***	.09	.19*	−.01	—												
10. Bedtime_F	.22**	.36***	.16*	.17	.22**	.37***	.19**	.27**	.61***	—											
11. Title_M	.38***	.40***	.05	−.08	.23***	−.04	.01	.05	.24**	.20**	—										
12. Title_F	.39***	.56***	.09	.13	.03	.16	−.02	.27**	.03	.25***	.38***	—									
13. Author_M	.33***	.37***	.03	−.05	.19**	−.09	.04	−.05	.20**	.17	.69***	.39***	—								
14. Author_F	.33***	.54***	−.02	.13*	−.02	.10	.05	.21**	.05	.28**	.38***	.64***	.48***	—							
15. Read A_M	.20**	.16*	.04	−.22*	.05	−.13	.01	−.20*	.13	.04	.16*	.08	.22**	.12	—						
16. Read B_M	.21**	.22**	−.01	−.18*	.06	−.16*	.03	−.21**	.08	−.03	.18**	.15	.26**	.10	.89***	—					
17. Read A_F	.13	.17	.12	.09	.15	.10	−.01	−.05	−.02	−.05	.06	.12	.23*	.26**	−.08	−.10	—				
18. Read B_F	.14	.16*	.09	.04	.09	.07	−.06	−.01	−.06	−.04	.05	.15	.22*	.30***	−.05	−.07	.93***	—			
19. Child R_G1a	.07	.17*	−.10	.01	−.04	.07	−.12	−.02	−.02	−.02	.08	.22***	.07	.31***	.05	.05	.29**	.29**	—		
20. Child R_G1b	.08	.15	−.09	.03	−.08	.05	−.12	−.04	−.02	−.04	.05	.23**	.06	.29***	.05	.06	.26*	.27*	.94***	—	
21. Child R_G3a	−.03	.08	−.07	.01	−.06	.06	−.03	−.13*	−.09	−.11	.03	.08	.03	.17*	.09	.05	.22**	.22**	.61***	.62***	—
22. Child R_G3b	−.02	.11	−.03	.02	−.03	.06	−.02	−.14*	−.05	−.05	.04	.12	.04	.21**	.11	.01	.26***	.28***	.62***	.63***	.93***

Correlations estimated with Full Information Maximum Likelihood in Mplus. Author, authors checklist; Bedtime, reading at bedtime; Child R, Child Word Reading Fluency; Extra R, reading at other times than at bedtime; F, fathers; Listen R, asking the child to read aloud; M, mothers; Read A & B, Word Reading Fluency Parent (Forms A & B); Spontaneous R, helping the child read words in daily life (e.g., cereal box); "Title, titles checklist. ***p < .001, **p < .01; *p < .05.

### Preliminary findings

To examine the children's word reading development, we modeled a latent change score from Grades 1 to 3. The latent change score model of word reading showed significant variation in Grade 3 (*σ* ^2^ = 293.810, *p* < .001) and significant change with a significant variation between Grades 1 and 3 (*M* = 42.061, *σ* ^2^ = 171.758, *p* < .001). The correlation between Grade 3 reading and the change in reading between Grades 1 and 3 was significant (*r* = .702, *p* < .001). This model had an excellent fit to the data (*χ* ^2^ (2) = 4.638, *p* = .098, CFI = 0.998, SRMR = 0.020, RMSEA = 0.074 [90% CI: 0.000–0.164]).

Further, in a confirmatory factor analysis, we estimated two latent variables (one for mothers and one for fathers), each reflecting the frequency of the three literacy activities in which the parents reported engaging with their child (see supporting information for details). Moreover, we estimated a second‐order factor reflecting the literacy resources at home by using first‐order factors of mothers' checklists, fathers' checklists, and the number of books at home as indicators. Both the mothers' and fathers' checklist factors were reflected by the title and author checklists. The books at home factor reflected both mothers and fathers' reported number of books at home. A Satorra‐Bentler chi‐square difference test confirmed scalar invariance for the specific factors for mothers' and fathers' activities (*χ* ^2^ (4) = 1.033, *p* = .905), and the model fitted the data well (*χ* ^2^ (51) = 72.442, *p* = .026, CFI = 0.956, SRMR = 0.065, RMSEA = 0.045 [90% CI = 0.016–0.067]).

### Intergenerational impact on child word reading

**Figure 1:**
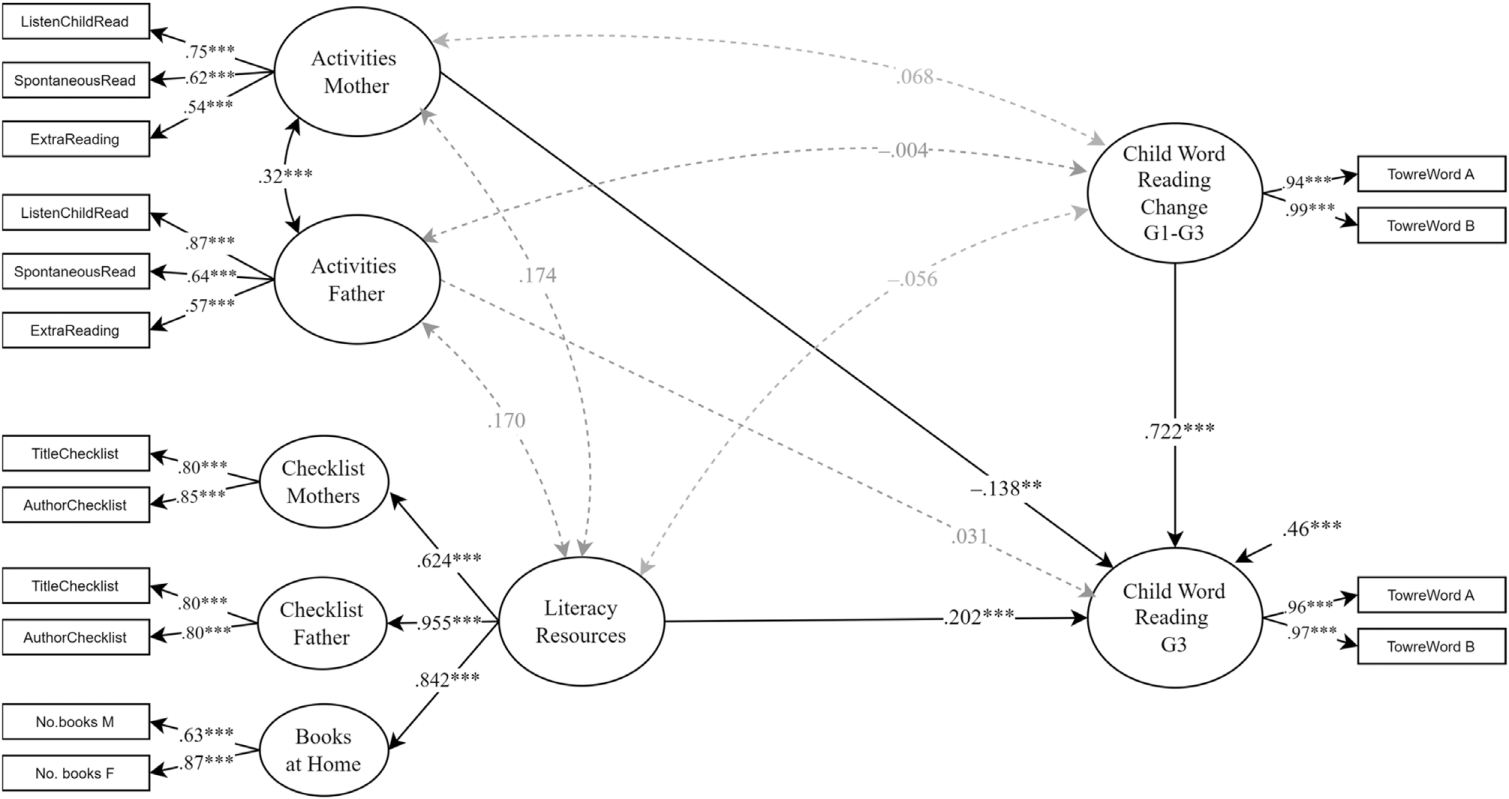
The home literacy environment predicting child word reading, not controlled for parental reading ExtraReading, reading at other times than at bedtime; F, fathers; ListenChildRead, asking the child to read aloud; M, mothers; SpontaneousRead, helping the child read words in daily life (e.g., cereal box); TowreWord = Word Reading Fluency Children (Forms A & B); G1 = Grade 1; G3 = Grade 3; ***p < .001, **p < .010, *p < .05

Initially, we examined the effect of the parents' reading activities and literacy resources on children's word reading without controlling for the effect of parental reading ability. To examine how the children's literacy environment was associated with reading, we regressed reading in Grade 3 on mothers' literacy activities, fathers' literacy activities, and literacy resources at home and the rate of change in reading between Grades 1 and 3. As can be seen in Figure 1, the word reading in Grade 3 was strongly predicted by the rate of change between Grades 1 and 3 (*β* = .772). Word reading in Grade 3 was negatively associated (*β* = −.138) with mothers' literacy activities and positively associated (*β* = .202) with the literacy resources at home. None of the variables were significantly correlated with the rate of change between Grades 1 and 3. There was a moderate correlation between the literacy activities of mothers and fathers but no significant correlation between literacy activities and the literacy resources at home. This model fitted the data very well (*χ* ^2^ (95) = 111.705, *p* = .116, CFI = 0.990, SRMR = 0.062, RMSEA = 0.027 [90% CI = 0.000–0.045]).

**Figure 2:**
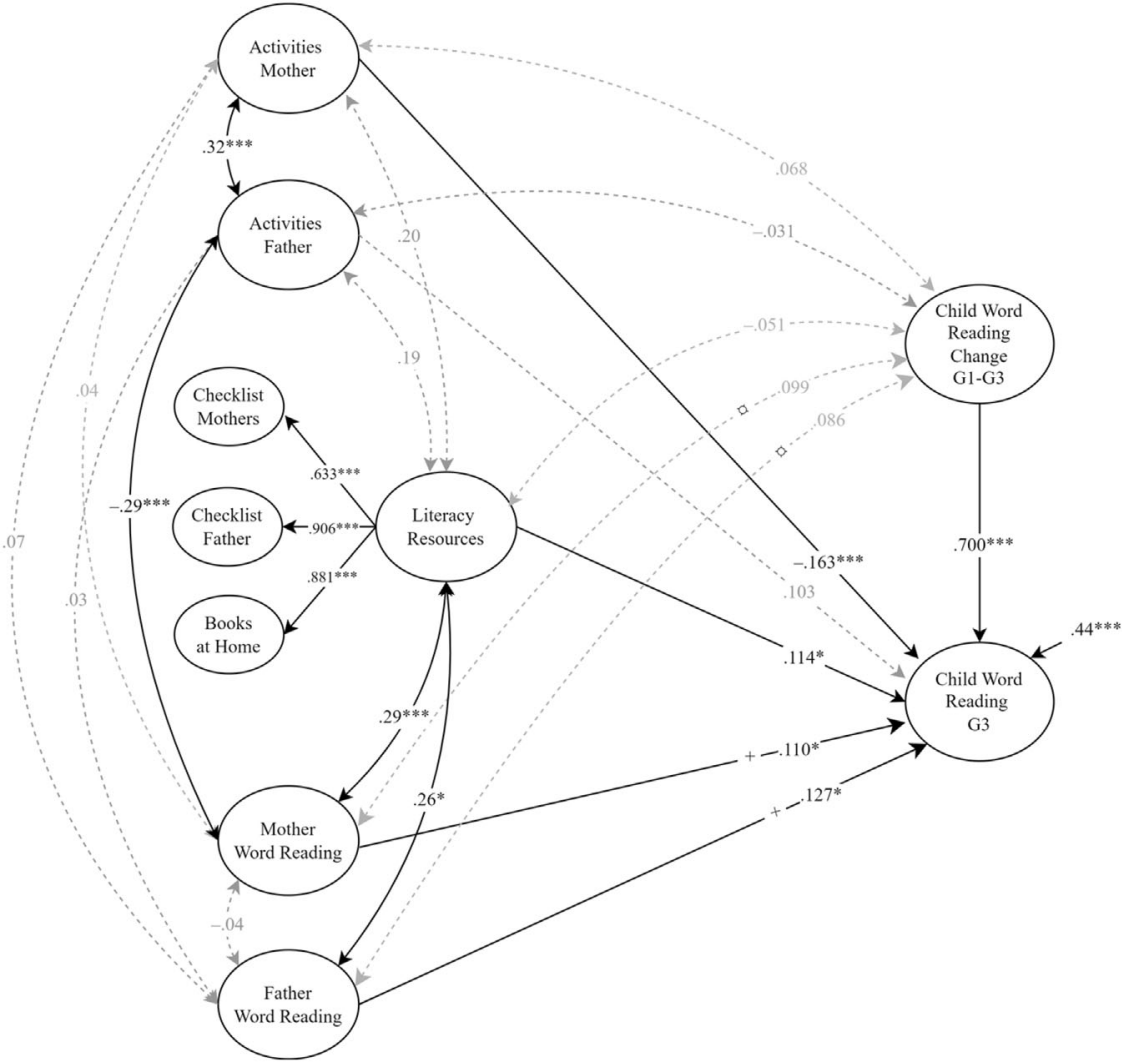
The home literacy environment predicting child word reading, controlled for parental reading For clarity, the measurement model with factor loadings for each construct was omitted from the diagram; ***p < .001, **p < .010, *p < .05; regression paths intersected with equal symbols were constrained to be equal.

Furthermore, we proceeded to examine the effect of parents' reading activities and literacy resources after accounting for parental reading abilities. In Figure 2, we can see that both mothers' literacy activities (*β* = −.163) and literacy resources at home (*β* = .114) were still associated with children's reading skills in Grade 3 after controlling for the reading skills of both mothers and fathers. In addition, mothers' and fathers' reading skills predicted the children's reading skills in Grade 3 (*β* = .110 and .127 for mothers and fathers, respectively). None of the variables were significantly correlated with the rate of change between Grades 1 and 3. Following the familial control method (Hart et al., 2021), we constrained the unstandardized regression paths from mothers' and fathers' word reading to children's word reading in Grade 3 to be equal to represent the 50% reading liability each parent passes on to the child. In addition, the correlations between both parents' reading and the rate of change were constrained to be equal between parents. Our data fully supported these assumptions of equality (Wald test: *χ* ^2^ (2) = 1.586, *p* = .452; the standardized coefficients can still differ as the variances can vary). Lastly, the model showed moderate significant correlations between mothers and fathers' word reading and the literacy resources at home. In contrast, mothers' word reading was negatively correlated with fathers' reading activities, but no significant correlations were found between each parent's word reading and the frequency of their reading activities with their child. This model had a very good fit to the data (*χ* ^2^ (154) = 181.789, *p* = .062, CFI = 0.987, SRMR = 0.061, RMSEA = 0.027 [90% CI = 0.000–0.042]).

Our results indicate that mothers' reading activities and the literacy resources at home predict child word reading in Grade 3 beyond the effect of parental reading. Parental reading predicted children's word reading in Grade 3 independent of the measures of the reading environment in Grade 2 and the growth of reading between Grades 1 and 3.

## Discussion

In this study, we examined the intergenerational impact of mothers and fathers on the development of children's word‐reading abilities through literacy resources and reading activities at home. Our results suggest that the HLE (created by both parents) significantly predicts children's reading skills in third grade, even when accounting for parental reading skills as a proxy for genetic confounding. Parental reading skills significantly predicted children's word reading ability in Grade 3. All these associations persisted after controlling for children's growth in reading skills between Grades 1 and 3.

Our study suggests that the intergenerational impact of parents on their children is, in part, through home literacy resources. The home literacy resources were manifested through parents' knowledge of children's literature and estimates of the number of children's books at home. Our analyses revealed that parents' contributions to home literacy resources reflect a common latent construct (see Figure 1). This latent construct of home literacy resources significantly predicted differences in children's word reading ability in Grade 3. Importantly, even after controlling for the confounding effect of parental reading ability, the impact of home literacy resources on children's reading remained significant, albeit attenuated (see Figures 1 and 2; from .20 to .11). These findings replicate the results from studies with similar genetic confounding control (van Bergen et al., 2017; Zhang, Inoue, & Georgiou, 2024). The attenuation of the effect of home literacy resources on reading is consistent with home literacy resources reflecting a combination of genetic transmission (as captured by parental skills) and environmental transmission. As discussed by van Bergen et al. (2017), the environmental transmission, or environmental effect, of home literacy resources could just reflect the importance of the availability of books. Alternatively, the availability of books could stand for something larger, such as parents' broader world knowledge and interest that they share with their children.

Furthermore, a significant predictor of children's word reading in Grade 3 beyond parental reading skills was the frequency with which mothers, but not fathers, teach reading (Figures 1 and 2; Activities Mother). Interestingly, that this association was negative suggests that mothers read with or to children more often when they struggle. Indeed, previous research from transparent orthographies has demonstrated a shift in the relationship between parental reading activities and children's reading skills from positive in kindergarten to negative in Grade 1 (Silinskas et al., 2012) and a persistent negative association through Grade 2 (Silinskas et al., 2013). The Norwegian orthography is more transparent than English; hence, after 1 year of reading instruction, Norwegian children can already decode most words (Seymour, Aro, & Erskine, 2003). This may explain why mothers reduce their involvement in reading activities as children become proficient readers and why we see this negative association more in transparent orthographies like Finnish and Norwegian compared to in the less transparent English orthography.

Conversely, fathers' reported frequency of reading activities was not predictive. The scarcity of studies focusing on fathers' contributions to the reading environment limits our ability to compare results. An exception is a study examining the separate contributions of mothers and fathers, where fathers' frequency of reading activities predicted child word reading only when mothers had less than a bachelor's degree (Foster, Froyen, Skibbe, Bowles, & Decker, 2016). We could not investigate this, as our sample predominantly consisted of highly educated parents. Interestingly, the frequency of fathers' reading activities was negatively associated with mothers' reading skills. This suggests that fathers' engagement—although not significantly associated with children's reading—may serve as a compensatory factor for mothers' reading ability.

Parental reading ability emerged as an independent predictor of word reading at Grade 3, beyond the effect of early word reading. This aligns with the findings by Khanolainen et al. (2024), suggesting that shared genes between parents and children continue to impact not only beginning reading skills but also later reading development. This fits with a growth‐modeling twin study that showed that shared‐environmental influences were stable, while genetic influences changed between early and later reading skills (Logan et al., 2013). Together, these longitudinal studies reinforce that parents transmit a disposition for learning to read, which influences not only early reading skills but also later reading development.

In conclusion, our investigation underscores the intergenerational transmission of literacy skills. Even after accounting for parental reading, children's reading was significantly predicted by home literacy resources and parental reading instruction. This study also suggests that mothers' informal teaching of reading is independent of other aspects of the HLE. Moving forward, research in this domain should consider the evolving dynamics of several aspects of the HLE beyond the initial stages of schooling while also accounting for the stable confounding effect of genetics.

## Ethical considerations

Consent for the children's participation in the study was obtained from the parents for all the children. The study, including the Data Protection Impact Assessment (DPIA), was accepted by Sikt: Norwegian Agency for Shared Services in Education and Research.

**Key points**

- Parents influence their children's reading development not only by shaping the home literacy environment but also by passing down genetic factors. Consequently, correlations between the home literacy environment and children's reading are genetically confounded.
- To mitigate genetic confounding, we accounted for parental reading ability, known as the familial control method.
- Beyond parental reading ability, children's word reading is predicted by the literacy resources in the home and by mother–child reading activities.
- The mothers of children who struggle tend to engage in more reading activities. This might reflect an adaptive response to support children facing challenges in word reading.
- Home literacy resources, including the number of books and parents' knowledge of children's literature, may reflect the availability of learning opportunities.

## Supporting information


**Appendix S1.** Preliminary confirmatory factor analyses of the reading activities and literacy resources for mothers and fathers.

## Data Availability

The data and output files (Mplus and R Markdown files) are available at: https://osf.io/7qcr4/.
