# Supplementary material for: The intergenerational impact of mothers and fathers on children's word reading development
Source: J Child Psychol Psychiatry. 2025 Jan 2;66(7):946–55. doi: 10.1111/jcpp.14107 (PMC12198930; doi:10.1111/jcpp.14107)
Supplement: Supplementary file 1 — Appendix S1. Preliminary confirmatory factor analyses of the reading activities and literacy resources for mothers and fathers. [file JCPP-66-946-s001.docx]

# Supporting Information

## Appendix S1

## *Preliminary Confirmatory Factor Analyses of The Reading Activities and Literacy Resources for Mothers and Fathers*

First, we assessed the four indicators of reading activities for mothers and fathers. The two-factor model of activities had an inadequate fit to the data (χ^2^ (15) = 36.959, *p* = .001, CFI = .916, SRMR = .056, RMSEA = .084 [90% CI =.050–.119]). Our results showed that the indicator of the frequency of bedtime reading had relatively low factor loadings to the latent factor of activities for mothers (λ =.35) and fathers (λ =.41). Removing these indicators from the latent factors for mothers and fathers resulted in an excellent model fit (χ^2^ (5) = 3.314, *p* = .651, CFI = 1.00, SRMR = .025, RMSEA = .000 [90% CI =.000–.078]). Our results also showed scalar invariance for the latent factors of reading activities for mothers and fathers (Satorra-Bentler chi-square difference test*:* χ^2^ (4) = 1.106, *p* = .893), suggesting that parents interpreted the indicators equally. Therefore, the factor loadings of equal indicators and their intercepts were constrained to be equal between parents in subsequent analyses.

We then extended the two-factor model of reading activities with two latent indicators of literacy resources for mothers and fathers (e.g., title and authors checklists and the number of children’s books at home). The model fit of the four-factor model was χ^2^ (51) = 85.197, *p* = .002, CFI = .930, SRMR = .068, RMSEA = .057 (90% CI =.035–.078). The modification indices suggested dependencies between the recognition checklists for mothers and fathers and between the number of books reported by both parents. The correlation between latent factors of the literacy resources of mothers and fathers was moderate to high (*r* = .640, *p* < .001).

We addressed this by creating a latent factor of the number of books reported by both parents. The indicators of children’s book titles and author recognition were modelled separately for mothers and fathers, resulting in three latent factors of literacy resources. Finally, because the previous model showed high latent correlations of the literacy resources between parents, we added a common shared factor (second-order factor) for the literacy resources, thereby capturing the shared contribution among parents. This model had a good fit to the data (χ^2^ (51) = 72.442, *p* = .026, CFI = .956, SRMR = .065, RMSEA = .045 [90% CI =.016–.067]).

The final model consisted of two latent factors of reading activities and a second-order factor of the literacy resources (including title and author checklist for mothers and fathers, respectively, and the number of books at home reported by both parents).

We acknowledge that the most widely used model of HLE (Sénéchal & LeFevre, 2002) posits specific associations between the formal HLE and code-based skills and between the informal HLE and meaning-based skills. However, there is additional evidence that does not strictly follow this model. For instance, aspects of the informal HLE (e.g., number of books) have been significantly associated with code-based skills like reading fluency (van Bergen et al., 2017; S.-Z. Zhang et al., 2023). Hamilton et al. (2016) compared the effect of the formal and informal HLE and found that both forms of environment explained differences in code-based skills a year later. For this reason, we model the excluded indicator of “bedtime reading” as part of both the latent factor of reading activities and literacy resources to test all possibilities.

Our analyses show that the indicator of “bedtime reading” could not be modeled as literacy resources at home either. Poor fit to the data indicated that this was not an equally good indicator as the other indicators. In addition, the modification indices showed that the bedtime reading indicator had residual dependencies with several of the indicators of reading activities. As this this indicator was initially tested in the latent factor of reading activities with poor fit to the data, the bedtime reading indicator was excluded. For a reference, Hamilton et al. (2016) reported a similar issue, excluding bedtime reading and number of books at home from a latent factor of informal HLE, opting for a latent factor consisting only of recognition of checklist.
